# Role of the bicarbonate-responsive soluble adenylyl cyclase in pH sensing and metabolic regulation

**DOI:** 10.3389/fphys.2014.00042

**Published:** 2014-02-10

**Authors:** Jung-Chin Chang, Ronald P. J. Oude-Elferink

**Affiliations:** Tytgat Institute for Liver and Intestinal Research, Academic Medical Center, University of AmsterdamAmsterdam, Netherlands

**Keywords:** soluble adenylyl cyclase, carbonic anhydrase, vacuolar-ATPase, protein kinase A, pH regulation, apoptosis, barrier function

## Abstract

The evolutionarily conserved soluble adenylyl cyclase (sAC, adcy10) was recently identified as a unique source of cAMP in the cytoplasm and the nucleus. Its activity is regulated by bicarbonate and fine-tuned by calcium. As such, and in conjunction with carbonic anhydrase (CA), sAC constitutes an HCO^−^_3_/CO^−^_2_/pH sensor. In both alpha-intercalated cells of the collecting duct and the clear cells of the epididymis, sAC is expressed at significant level and involved in pH homeostasis via apical recruitment of vacuolar H^+^-ATPase (VHA) in a PKA-dependent manner. In addition to maintenance of pH homeostasis, sAC is also involved in metabolic regulation such as coupling of Krebs cycle to oxidative phosphorylation via bicarbonate/CO_2_ sensing. Additionally, sAC also regulates CFTR channel and plays an important role in regulation of barrier function and apoptosis. These observations suggest that sAC, via bicarbonate-sensing, plays an important role in maintaining homeostatic status of cells against fluctuations in their microenvironment.

## Introduction

Regulation of intracellular pH (pH_i_) plays an important role in maintaining physiologic processes in living cells because most enzymes function properly only under a narrow window of pH. The cytoplasm is constantly acidified by cellular metabolism and the negative resting membrane potential also favors influx of protons; this poses a constant challenge to the maintanence of cytosolic pH. Homeostasis of intracellular pH depends on the buffering capacity of intracellular bicarbonate concentration, other weak acids/bases and macromolecules on one hand. On the other hand, a spectrum of membrane transporters (ion channels and pumps) responds instantaneously to fluctuation of pH_i_ and extracellular pH (pH_o_) in order to actively maintain pH_i_ homeostasis (Roos and Boron, 1981; Boron, 2004). In eukaryotic cells, the endomembrane system is also equipped with channels and pumps to maintain a different pH inside individual compartments for optimal functioning. Much has been explored as to how the cellular machinery reacts during the fluctuation of intracellular or extracellular pH; however, the mechanism by which cells sense the fluctuation of pH in order to react accordingly remains uncertain.

Strictly speaking, almost every cellular protein is sensitive to pH; however, to fulfill the role as a pH sensor, the candidate must be capable of detecting pH changes within the physiologically relevant range and initiating a signaling cascade to its effector to maintain homeostasis. For proper functioning, cells would ideally sense the changes of intracellular pH as a result of cellular metabolism or changes in extracellular pH. Whereas unicellular organisms dissipate their metabolic waste by diffusion and transport into their immediate environment, pH homeostasis in multi-cellular organisms relies on the circulation system and organs specialized in acid/base regulation. The logic of pH homeostasis is therefore different in these specialized organs.

A few notable examples would be the alpha-intercalated cells in the kidney, clear cells in the epididymis and the gill of dogfish shark. In these acid/base-regulating organs, specialized cells maintain not only the homeostasis of their own intracellular pH, but also the extracellular pH. In order to react timely to acute changes in extracellular pH and therefore intracellular pH, the sensor would be expected to exert its downstream effector in an extremely rapid fashion such as tuning the activity of existing effectors by post-translational modification, inserting the ready-made effectors or remove the counteractive ones by endocytosis, or coupling ion channels or pumps for higher efficiency. For chronic changes, the signaling may involve also transcriptional regulation.

Many potential acid/base sensors have been proposed with special attention paid to the kidney for its indispensable role in pH homeostasis. Potential candidates proposed thus far include acid/base-sensing receptors (GPR4, InsR-RR), pH-sensitive kinases with NHE3 as downstream effector (Pyk2/ETB), vacuolar H^+^-ATPase (V-ATPase) in endosome, pH-sensitive ion channels (ASICs, TASK, ROMK), and soluble adenylyl cyclase (sAC, ADCY10) (Brown and Wagner, 2012). In this review, we focus on recent evidence how sAC could serve as an intracellular pH sensor and how it maintains pH homeostasis, mainly through the V-ATPase. In addition, we highlight other (proposed) functions of sAC that appear secondary to changes in local bicarbonate concentrations.

## Soluble adenylyl cyclase (sAC) is an evolutionarily conserved bicarbonate sensor

sAC is a special member of the mammalian adenylyl cyclase family that generates one of the most universal second messenger cAMP from ATP. Its activity was first described as Mn^2+^-dependent, LH- and FSH-insensitive in the cytosolic fraction of adult rat testis homogenate, a surprising finding at that time in that the canonical mammalian adenylyl cyclase activity was known to come solely from the plasma membrane (Braun and Dods, 1975). Unlike its transmembrane counterparts, sAC is not stimulated by the transmembrane adenylyl cyclase (tmAC) stimulator fluoride and forskolin, nor is it regulated by G-proteins (Buck et al., 1999). Rather, it is stimulated by bicarbonate and its activity is independent of pH (Chen et al., 2000). Structurally, sAC and tmACs are monomeric proteins and catalyze cAMP production through dimerization of the two catalytic domains (Kamenetsky et al., 2006). They share the homology in their two catalytic domains, but sAC lacks the 2 hydrophobic domains (each with 6 predicted membrane-spanning helices) of tmACs (Hurley, 1999; Steegborn et al., 2005b), which explains the cytoplasmic localization of sAC. Phylogenetic analysis of the catalytic domains revealed that sAC is evolutionarily conserved across metazoans and bacteria (Kobayashi et al., 2004). Like tmAC, sAC belongs to Class III nucleotidyl cyclase, but surprisingly, sAC is most closely related to the cyanobacterial (*Anabaena spirulensis* and *Spirulina platensis*) and myxobacterial (*Stigmatella aurantiaca*) adenylyl cyclases, whose activity can also be stimulated by bicarbonate (Yan et al., 1998; Chen et al., 2000; Cann et al., 2003).

In the human genome, the single-copied gene ADCY10 encodes a 187 kD full-length enzyme (Geng et al., 2005) as in rats, which is substantially larger than the more abundant, truncated isoforms that were originally purified from rat testis(~50 kD). The truncated sAC (tsAC) was then proposed to be the proteolytic product of the full-length protein. Later, functional analysis of splice variants from germ cell sAC in mice and rats revealed another possible mechanism where deletion of exon 13 (NCBI RefSeq NM_173029.3) by alternative splicing introduces an in-frame stop codon and terminates the open reading frame prematurely to yield a 20-fold more active 48 kD truncated enzyme (Buck et al., 1999; Jaiswal and Conti, 2001). The tsAC contains almost exclusively the two conserved catalytic domains (C1 and C2) while the full-length enzyme has an additional non-catalytic C-terminal part, that harbors an autoinhibitory domain that accounts for the reduced bicarbonate-stimulated activity in full-length enzyme (Chaloupka et al., 2006). The physiologic function of this domain in the full-length enzyme is as yet unknown.

Two sAC knockout mouse models exist. The initial construct to generate a sAC knockout mice by homologous recombination was designed to replace exon 2–4 (NCBI RefSeq NM_173029.3) of catalytic domain C1 in the wild-type sAC locus with an in-frame IRES-lacZ expression cassette. This resulted in a mouse model with complete sAC disruption only in germ cells (Sacy^tm1Lex/^Sacy^tm1Lex^, sAC C1KO). The sAC C1KO mice display male infertility (Esposito et al., 2004). Extensive phenotypic analysis revealed that female mice have increased circulating cholesterol and triglyceride and that both male and female mice have slightly elevated heart rates [as deposited in the Mouse Genome Database (Eppig et al., 2005)]. More recently, it was reported that an alternative start site upstream of exon 5 is used by somatic cells to produce yet another isoforms containing only one catalytic unit C2. This isoforms is unaffected by the C1KO (Sacy^tm1Lex/^Sacy^tm1Lex^) gene disruption (Farrell et al., 2008). Another recently established sAC knockout model is generated with the LoxP-Cre system by targeting the exon 9–11 (NCBI RefSeq NM_173029.3) of catalytic domain C2 (sAC C2KO). The sAC C2KO mice also display male infertility while the detailed phenotypic characterization and the confirmation for a total knockout is pending (Chen et al., 2013).

sAC is highly expressed in male germ cells and is widely expressed at lower level in almost all tissues thus far examined. sAC is located in several subcellular compartments, including the cytosol, the nuclei (Zippin et al., 2004), and the mitochondria (Acin-Perez et al., 2009; Valsecchi et al., 2013). In both mitochondria and the nucleus, the elements for the whole signaling cascade sAC-cAMP-protein kinase A (PKA) are present. In addition, sAC has been shown to co-localize with microtubules, centrioles, and mid-body (Zippin et al., 2003), essential structures involved in scaffolding, vesicular trafficking, and cell division. In the presence of various isoforms of CA in the cytosol (CAII), the plasma membrane (CAVI), and mitochondria (CA VA and CA VB) (Supuran, 2008), changes in carbon dioxide (CO_2_) tension and proton concentration are rapidly equilibrated via the reaction: CO_2_+H_2_O↔H_2_CO_3_↔HCO^−^_3_+H^+^ and are reflected in bicarbonate concentration, which is then sensed by sAC in different intracellular compartments. With the scaffolding A-kinase anchoring protein (AKAP) (Wong and Scott, 2004), the cAMP effectors, PKA and Epac (**e**xchanger **p**rotein directly **a**ctivated by **c**AMP), and their local substrates are organized into individual microdomains where associated phosphodiesterases (PDEs) limit cAMP diffusion by degrading it into AMP (Zaccolo and Pozzan, 2002; Zaccolo et al., 2002; Mongillo et al., 2004). Phosphorylation of local substrates by PKA is also counterbalanced by associated protein phosphatase-1 (PP-1) (Schillace and Scott, 1999; Schillace et al., 2001). The non-selective messenger cAMP is hereby able to specifically coordinate physiologic processes within individual microdomains.

sAC distinguishes itself from other pH sensor candidates in that its activity is stimulated by physiologic concentration of bicarbonate (via *V*_max_, *EC*_50_ = 25.4 ± 7.6 mM) but not pH (Chen et al., 2000) and is fine-tuned by micromolar calcium (via *K*_m_, *EC*_50_ ≈ 750 μM) (Jaiswal and Conti, 2003; Litvin et al., 2003), another ubiquitous second messenger. Upon stimulation, sAC generates the ubiquitous second messenger cAMP, whose effector proteins in mammalian cells include PKA, Epac (Gloerich and Bos, 2010), and cyclic nucleotide-gated ion channels (CNGs and HCNs) (Kaupp and Seifert, 2002). The sAC-dependent cAMP has been shown to activate PKA (Sample et al., 2012), which is able to respond in both short-term and long-term manners. PKA can phosphorylate its target proteins in response to acute change, while translocation of its catalytic unit into the nuclei results in phosphorylation of the cAMP response element binding protein (CREB) and transcriptional regulation of genes with cAMP response element (CRE) in their promoter region (Mayr and Montminy, 2001). Potential targets that are involved in direct acid/base regulation include SLC4A2, CFTR, ATP1A1, ATP6V0B, ATP6V0D1, ATP6V1D, ATP6V1A, ATP6V1B2, and ATP6V0C (Zhang et al., 2005). For extensive review of cAMP signaling by sAC, readers are referred to Tresguerres et al. (2011).

pH-sensing via bicarbonate has two advantages over via direct proton-sensing. Firstly, although diffusion of proton in the pure water (*D*_H+_ = 9.311 × 10^−5^ cm^2^/s at 25°C) is much faster than that of bicarbonate (*D*_bicarbonate_ = 1.185 × 10^−5^ cm^2^/s at 25°C) and CO_2_(*D*_CO2_ = 1.91 × 10^−5^ cm^2^/s at 25°C) (Vanysek, 2005), diffusion of protons in the cytosol (*D*_H+_ = 8.2 × 10^−7^ ~ 1.2 × 10^−6^ cm^2^/s at 37°C under CO_2_/HCO^3−^-free condition) is much slower due to repeated association and dissociation with macromolecules (Zaniboni et al., 2003). In comparison, the diffusion of bicarbonate and CO_2_ are affected to a lesser degree (*D*_bicarbonate_ = 8.7 × 10^−6^ cm^2^/s and *D*_CO2_ = 1.59 × 10^−6^ cm^2^/s at physiological ionic strength and 25°C) (Gros et al., 1976). Moreover, the apparent diffusion coefficient for proton (*D*^app^_H_) in cytosol has been determined to be 2–5-fold higher in the presence of physiologic CO_2_/HCO^3−^ buffering compared to that in the absence of CO_2_ (Stewart et al., 1999; Spitzer et al., 2002; Zaniboni et al., 2003), which implies that proton diffusion is facilitated via carbonic shuttle and that the pH gradient is mitigated in the presence of CAs. Detecting pH through the translated index, bicarbonate, is thus advantageous in that cells can sense the fluctuation in pH timely and safely while maintaining pH homeostasis.

Secondly, while glycolysis results in anion and proton load, the end product of the Krebs cycle in mitochondria is CO_2_ and H_2_O. In the presence of CA5A and CA5B, CO_2_ production from the Krebs cycle, an activity index for aerobic respiration, is specifically and readily translated into bicarbonate and subsequently sensed by sAC. As will be discussed in a later section, bicarbonate sensing by mitochondrial sAC plays an important role in coupling the Krebs cycle to respiratory chain efficiency and therefore make good use of the reducing equivalents and prevent excessive formation of reactive oxygen species (ROS). Hence, sAC is as much a metabolic sensor as it is an indirect pH sensor. For the summary of the functions of sAC, please refer to the **Appendix**.

## sAC is indispensable in spermatogenesis and fertilization

Expression of sAC mRNA in mice is first detectable in mid-pachytene spermatocyte in meiotic prophase and becomes much more abundant in round spermatids (Sinclair et al., 2000). Similarly, Mn^2+^-sensitive adenylyl cyclase activity (Braun and Dods, 1975) and full-length sAC (Xie and Conti, 2004), are detected first in testis homogenate from rats aged 20–25 days, corresponding to the secondary spermatocyte stage. The adenylyl cyclase activity and protein expression level of sAC increase further and plateau as the sperms become mature. Northern blot analysis of poly-A^+^ mRNA from preparations of adult mouse testis reveals that the sAC activity originate from germ cells but not Sertoli or Leydig cells (Sinclair et al., 2000). Although mRNA of sAC is detectable by RT-PCR in ovarian preparations, Northern blot and *in situ* hybridization reveal only background staining, which implies that the high abundance of sAC expression is not a general phenomenon for all germ cells but specific for males (Sinclair et al., 2000).

Because of its predominant expression in male germ cells, the discovery of a sAC-dependent cAMP pool sheds new light on spermatogenesis and fertilization. It is known that the acidic microenvironment in epididymis (pH 6.5~6.8) (Levine and Marsh, 1971; Levine and Kelly, 1978) is required for maturation of spermatozoa and keeping the spermatozoa in dormancy. Before becoming capable of fertilizing the egg, spermatozoa must first undergo a series of bicarbonate-induced (Lee and Storey, 1986; Boatman and Robbins, 1991) and cAMP-dependent (Vijayaraghavan and Hoskins, 1985, 1986; Visconti et al., 1999) processes, including alteration in plasma membrane fluidity (Flesch et al., 2001), protein tyrosine phosphorylation (Visconti et al., 1995a,b), and acquisition of hypermotility pattern (Vijayaraghavan et al., 1985) while they transit from the vas deferens to the fallopian tube. These events are collectively referred to as “capacitation,” the molecular mechanism of which is not completely characterized. The capacitation process commences during ejaculation as the spermatozoa leave the epididymis and mix with the acidic prostatic fluid and alkaline seminal vesicle fluid. Neutralization by the alkaline seminal vesicle fluid (pH 7~8) (Huggins et al., 1942; Owen and Katz, 2005) triggers the hypermotility of sperms. The capacitating events continue as spermatozoa transit though the female reproductive tract. The identification of sAC as a bicarbonate sensor and a unique cytosolic source of cAMP in spermatozoa provides a mechanism how the dormant sperms sense this long-known environmental cue and become capacitated.

Studies of mice with gene disruption at the C1 catalytic unit (sAC-C1KO) revealed that the predominant pool of cAMP in spermatozoa is produced by sAC (Esposito et al., 2004; Xie et al., 2006) and there is a dose-dependent effect of genetic ablation of sAC on bicarbonate-stimulated adenylyl cyclase activity in murine spermatozoa. The sAC-C1KO mice show no gross abnormalities and develop apparently normal testes and epididymides. However, the male mice are infertile (Esposito et al., 2004). Their sperms are characterized with flagellar angularity and impaired motility. Under capacitation condition, the sAC-C1KO sperms show aberrant tyrosine phosphorylation pattern and are incapable of fertilizing eggs *in vitro*. Bypassing sAC by addition of membrane-permeant cAMP analog (dibutyryl-cAMP and cAMP•AM) induced some progressive motility in the sAC-C1KO sperms; however, careful examination revealed that the capacitation-associated hyperactive status remains absent and the aberrant tyrosine phosphorylation pattern is not fully corrected. Furthermore, the sAC-C1KO sperm failed to fertilize eggs *in vitro* even if they are incubated with capitation buffer supplemented with the membrane permeable cAMP analog (Hess et al., 2005; Xie et al., 2006).

The discrepancy between the wild-type sperms and the permeable cAMP analog-rescued sAC-C1KO sperms points out the role of sAC in the spermatogenesis and maturation of spermatids in the epididymis, which correlates with its progressive expression in the second half of spermatogenesis. This is best evidenced by study with KH7, a sAC-specific inhibitor. KH7-treated wild-type spermatozoa have significantly lesser bicarbonate-induced cAMP production, reduced motility and become incapable of fertilize eggs *in vitro*. However, in contrast to sAC-C1KO sperms, addition of the membrane-permeant cAMP analog to KH7-treated sperms completely rescues the motility and *in vitro* fertilization capability. These experiments provide important evidence that sAC is not only important for capacitation and fertilization but also indispensable for spermatogenesis (Hess et al., 2005). As discussed in next section, sAC in specialized epididymal epithelial cells plays an important role in the pH regulation of epididymal fluid and further contributes to spermatozoa maturation.

## sAC regulates extracellular pH by mobilizing vacuolar H^+^-ATPase in specialized epithelia

Vacuolar H^+^-ATPases (V-ATPase) comprise a family of multi-subunit ATP-dependent proton pumps that are organized into two domains: the 650 kD cytosolic V_1_ domain and the 260 kD membrane-embedded V_0_ domain. Using the energy derived from ATP hydrolysis by the cytoplasmic V_1_ domain, protons are transported via the rotatory machinery in the integral V_0_ domain from the cytosol into the lumen of organelles or to the extracellular space (Nishi and Forgac, 2002; Forgac, 2007; Toei et al., 2010). ATPases are expressed ubiquitously in the endomembrane system, where it controls luminal pH of each compartment along the exocytic and endocytic pathways to maintain appropriate pH locally for specific physiologic processes (Mellman et al., 1986; Hinton et al., 2009). In specialized CA- and mitochondria-rich epithelial cells, V-ATPases are highly expressed and cycle between the apical plasma membrane and subapical vesicles in response to changes of extracellular pH (Brown and Breton, 1996). Apical accumulation of V-ATPase in these specialized epithelial cells is correlated with increased proton secretion (Steinmetz, 1986; Stetson and Steinmetz, 1986). The high expression of CA in these proton-secreting epithelia suggests that bicarbonate, as a translated pH index, is a key signal for proton secretion. This point of view is supported by recent investigations in clear cells in epididymis, alpha-intercalated cells in kidney and V-ATPase-rich cells in the gill of dogfish where sAC is highly expressed (Pastor-Soler et al., 2003; Paunescu et al., 2008; Tresguerres et al., 2010), functions as a bicarbonate sensor and maintains extracellular pH homeostasis by regulating V-ATPase vesicular trafficking through HCO^−^_3_/sAC/PKA signaling pathway. Here we discuss the HCO^−^_3_-sAC-PKA-V-ATPase pathway of clear cells in epididymis and alpha-intercalated cells in kidney.

### Clear cells in epididymis

Epididymis is formed by a highly convoluted tubule where the lumen stores spermatozoa and the luminal epithelial lining actively maintains a low-bicarbonate (2–7 mM) and acidic environment, which is critical for sperm maturation and quiescence before being ejaculated. Homeostasis of the acidic environment is achieved by cross-talk between the four different types of cells: principal, basal, narrow, and clear cells (Shum et al., 2009, 2011). Principal and basal cells are present throughout the epididymal epithelium, while narrow cells are located only in the initial segment and clear cells in the capus, the corpus, and the cauda of the epididymis. Importantly, principal cells reabsorb significant amounts of bicarbonate in the initial segment and caput epididymis via the apical sodium-proton exchanger NHE2 and NHE3, the basolateral anion exchanger AE2 and the sodium-bicarbonate cotransporter NBCe1 (Liu et al., 2012), while clear cells further acidify the luminal fluid via V-ATPase-mediated proton secretion (Da Silva et al., 2007).

Epididymal clear cells express abundant V-ATPase (Brown et al., 1992). Their apex possesses well-development microvilli and subapical vesicles (Brown and Montesano, 1980, 1981). Using *in vivo* perfusion of rat distal cauda epididymidis as a model, Pastor-Soler et al. demonstrated that the bicarbonate-sensing sAC, together with CAs, plays an important role in apical accumulation and endocytosis of V-ATPase in clear cells (Pastor-Soler et al., 2003). While most V-ATPases are co-localized with horseradish peroxidase-labaled endosome when perfused with phosphate-buffered saline (PBS) at pH 6.5, elevating the perfusate pH to 7.8 induces V-ATPase apical insertion and microvilli elongation. Inhibition of sAC by 2-hydroxyestradiol, a relative sAC-selective inhibitor (Steegborn et al., 2005a), prevents the alkaline-induced apical translocation of V-ATPases. Inhibition of CA by acetazolamide, a broad-spectrum inhibitor of CA, also decreases alkaline pH-induced apical accumulation of V-ATPase, underscoring its role in translating pH into bicarbonate concentration.

To unequivocally demonstrate that bicarbonate mediates the alkaline-induced apical translocation of V-ATPase, the authors perfused caudal epididymis at pH 7.1 with PBS or modified Hanks buffer containing 12 mM bicarbonate at 5% CO_2_. V-ATPases were located in subapical vesicles when perfused with PBS but translocated to apical membrane when perfused with Hanks buffer. The addition of 2-OH estradiol abolishes the bicarbonate-induced apical translocation of V-ATPase (Pastor-Soler et al., 2003). Using PKA-selective cAMP analog *N*^6^-monobutyryl-3′-5′-cAMP (6-MB-cAMP), Epac-selective 8-pCPT-2-*O*-Me-cAMP and PKA inhibitor myristoylated-PKI (mPKI), PKA was shown to be the responsible cAMP effector (Pastor-Soler et al., 2008).

The alkaline pH-induced V-ATPase membrane accumulation is further regulated by gelsolin and AMP-activated protein kinase (AMPK). Gelsolin, an actin-severing protein, is highly expressed in professional V-ATPase-rich proton-secreting cells, including clear cells, intercalated cells in kidney and osteoclasts. It facilitates membrane insertion of V-ATPase vesicles by favoring the actin cytoskeleton in a depolymerized status, which is counteracted by the actin-polymerizing agent Jasplakinolide (Beaulieu et al., 2005).

AMPK is a ubiquitously expressed and highly conserved metabolic sensor that is activated by increased [AMP]/[ATP] ratio. Under metabolic stress where energy consumption is greater than regeneration, AMPK is phosphorylated at Thr-172 in its α-subunit by its upstream kinases and favors catabolic processes over anabolic processes (Hardie et al., 2003; Hardie, 2008). Consistent with its role, activation of AMPK by AMP-analog activator 5-amino-imidazole-4-carboxamide-1-β-d-ribofuranoside (AICAR) or by the non-nucleotide activator A-769662 counteracts both alkaline pH-induced and 6-MB-cAMP-induced V-ATPase translocation and reduces the microvilli area of clear cells to a level that is lower than the baseline. Knockdown of AMPK α-subunit results in increased PKA phosphorylation of V-ATPase A subunit (ATP6V1A), the ATP hydrolytic site in the cytoplasmic V_1_ domain (Hallows et al., 2009) and a shared phosphorylation target of PKA and AMPK (Tuerk et al., 2007; Alzamora et al., 2010, 2013).

### Alpha-intercalated cells in kidney

In mammals, pH homeostasis is achieved by controlling the two components of the HCO^−^_3_/CO_2_ buffering system. The lungs ventilate “the volatile acid” CO_2_ while the kidneys dispose non-volatile acids, secrete proton and reabsorb bicarbonate. Alpha-intercalated cells are specialized proton-secretion cells in the collecting duct in kidneys. Like clear cells in epididymis, alpha-intercalated cells originate embryologically from the urogenital ridge, possess abundant subapical vesicles and are equipped with well-developed microvilli on the apex (Madsen and Tisher, 1986).

sAC is not only highly expressed in alpha-intercalated cells but also associated with V-ATPase as observed by immunofluorescent staining and coimmunoprecipitation (Paunescu et al., 2008). In acetazolamide-treated rats, a model for chronic increased bicarbonate delivery to the collecting duct, the alpha-intercalated cells are enlarged and have higher V-ATPase expression and more elongated apical microvilli as compared to control rats (Bagnis et al., 2001). Similar to epididymal clear cells, cAMP stimulates apical accumulation of V-ATPase, villi elongation, and proton secretion in alpha-intercalated cells in a PKA-dependent way (Paunescu et al., 2010). Furthermore, activation of AMPK by AICAR in alpha-intercalated cells also counteracts PKA-mediated V-ATPase apical accumulation (Gong et al., 2010).

The regulatory mechanism of PKA and AMPK on V-ATPase membrane accumulation in alpha-intercalated cells has been recently characterized by Alzamora et al. (2010, 2013). Phosphorylation of V-ATPase A subunit at Ser-175 by is necessary for PKA-mediated V-ATPase apical insertion. When Ser-175 is mutated to the unphosphorylatable alanine (S175A), PKA-mediated apical accumulation of V-ATPase is prevented. Consistently, cells expressing the S175A mutant have decreased bafilomycin-sensitive, V-ATPase-dependent acid secretion into medium at baseline and under 6-monobutyryl-cAMP (6-MB-cAMP)/IBMX stimulation compared with cells expressing wild-type A subunit. Furthermore, when the Ser-175 is mutated to the phosphomimetic aspartate (S175D), baseline bafilomycin-sensitive acid secretion is increased to a level that is comparable with 6-MB-cAMP/IBMX stimulated, Ser-175- expressing cells and is not further stimulated with 6-MB-cAMP/IBMX (Alzamora et al., 2010). On the other hand, AMPK prevents apical accumulation of V-ATPase likely by phosphorylating V-ATPase A subunit at a different residue, Ser-384. When Ser-384 is mutated to unphosphorylatable alanine (S384A), cells have a higher extracellular acidification than that of those express the wild-type protein. Moreover, the increased acid secretion in cells expressing S384A is not inhibited by the AMPK activator AICAR (Alzamora et al., 2013). These mutagenesis experiments provide a new regulatory mechanism of V-ATPase vesicular trafficking by differential phosphorylation of its A subunit phosphorylation, with phosphorylation at Ser-175 by PKA enhancing and phosphorylation at Ser-384 by AMPK preventing apical accumulation.

This differential phosphorylation could serve as a feedback mechanism for the bicarbonate-sAC-cAMP-PKA signaling. When elevated extracellular pH is translated into bicarbonate by CA and detected by sAC, the bicarbonate-activated sAC converts ATP into cAMP at a higher rate that results in PKA-mediated V-ATPase apical accumulation. Meanwhile, within the microdomain, PDEs hydrolyze cAMP to AMP to restrict the cAMP signaling locally. The resultant increase in [AMP]/[ATP] ratio would activate AMPK which in turn phosphorylates the A subunit of V-ATPase at Ser-384 and thus counterbalances PKA by possibly increasing endocytosis or preventing apical insertion of V-ATPase. Further investigations are needed to elucidate the molecular mechanism how AMPK counteracts PKA-mediated V-ATPase apical accumulation.

## sAC as a metabolic sensor

The emergence of a unique cytosolic source of cAMP by sAC provides new insights for fruitful cAMP signaling research in hormonal regulation of metabolism. Several lines of evidence support the idea that bicarbonate-responsive sAC is not only a candidate for metabolic regulator via cAMP signaling, like tmAC, but also a metabolic sensor. Firstly, sAC's location in both the cytosol and mitochondria is ideal to detect metabolic signals from glycolysis, Krebs cycle and oxidative phosphorylation. Secondly, most cellular catabolic processes (e.g., glycolysis and fatty acid β-oxidation) yield intermediary metabolites that fuel the Krebs cycle to produce the water-soluble electron carrier NADH and CO_2_. Electrons from NADH, succinate oxidation (Krebs cycle), fatty acid-CoA (via acyl-CoA dehydrogenase), and glycerol-3-phosphate (via glycerol 3-phosphate dehydrogenase) are transferred though the respiratory chain to oxygen. In a series of redox reaction, the energy derived therefrom is stored as proton-motive force for subsequent generation of ATP from ADP and P_i_ by ATP synthase. Both CO_2_ from the Krebs cycle (after being converted to bicarbonate by CA) and ATP from oxidative phosphorylation (substrate for sAC) can serve as a metabolic signal input for sAC. Thirdly, sAC shares same effectors with tmAC both transcriptionally (e.g., CREB) (Montminy, 1997; Daniel et al., 1998; Mayr and Montminy, 2001) and post-translationally (e.g., PKA) (Pilkis and Claus, 1991; Pilkis and Granner, 1992), rendering itself a new player in the well-established cAMP signaling in glycolysis and gluconeogenesis. Furthermore, it has been demonstrated that the whole set of elements for CA-sAC-PKA-CREB signaling are present not only in cytosol/nucleus but also in mitochondria, implying that transcription of the mitochondrial genome is also subjected to cAMP regulation via CRE in the promoter of target genes (Lee et al., 2005; Ryu et al., 2005).

Recently, sAC was shown to be involved in metabolic regulation in hippocampal astrocytes (Choi et al., 2012), insulin-secreting β-cell of endocrine pancreas (Ramos et al., 2008; Zippin et al., 2013), and mitochondria of liver and brain (Acin-Perez et al., 2009, 2011a,b).

### sAC regulates glycogen breakdown, glycolysis, and lactate shuttle in astrocytes

In brain, astrocytes play an indispensable role in supporting the energy requirement of adjacent neurons. To fulfill the energy requirement of neurons during intense neuronal activation, astrocytes and neurons must communicate. Activation of neurons results in increased extracellular potassium ([K]_ext_) which depolarizes astrocytes and results in alkalization of pH_i_ of astrocytes via electrogenic sodium/bicarbonate cotransporter NBCe1 (SLC4A4) (Chesler, 2003). Hence, [K]_ext_, an index of neuronal activity, can be translated into bicarbonate and activate sAC in astrocytes. Choi et al. (2012) showed that not only elevation of [K]_ext_ but also transient deprivation of [glucose]_ext_ result in increased sAC-dependent, DIDS-sensitive cAMP concentrations in primary rat astrocyte culture (DIDS is an irreversible inhibitor of bicarbonate-dependent transport). Furthermore, in rat hippocampal slices, an elevation of [K]_ext_ or transient deprivation of [glucose]_ext_ induced glycogen breakdown and glycolysis in astrocytes as well as a lactate shuttle to adjacent neurons, which is prevented by sAC-specific inhibitors, KH7 and 2-hydroxyestradiol, and DIDS (Choi et al., 2012).

### sAC couples krebs cycle to respiratory chain in mitochondria

The mitochondrial respiratory chain is a metabolic convergence point where reducing equivalents (NADH and FADH_2_) produced in catabolic processes are utilized to generate ATP. Imbalance between the generation and the utilization would lead to excessive production of ROS that are harmful to the cell (Petrosillo et al., 2001; Li et al., 2003; Chen et al., 2007). The Krebs cycle is one of the main reducing equivalent feeders for respiratory chain. At each turn of the cycle, two CO_2_ are generated by oxidative decarboxylation in addition to three NADH molecules, one FADH_2_ and one GTP/ATP. CO_2_ is readily converted to bicarbonate by mitochondrial CA5A and CA5B and can serve as a metabolic signal for sAC.

Using mitochondria isolated from mouse liver and brain, Acin-Perez et al. demonstrated that a mitochondrial CO_2_-HCO^−^_3_-sAC-cAMP-PKA (mito-sAC) signaling cascade is responsible for coupling the Krebs cycle to the respiratory chain (Acin-Perez et al., 2009) with PDE2A2 serving as a restrictive regulator of the intra-mitochondrial cAMP pool (Acin-Perez et al., 2011a). Both direct stimulation by bicarbonate in a physiologic relevant range (10–40 mM) and CO_2_ generated by α-ketoglutarate dehydrogenase complex result in an increase of respiratory chain activity; this can be inhibited by KH7 and acetazolamide, indicating that conversion of CO_2_ to bicarbonate by mitochondrial CA is a critical step. Consistently, interruption of the mito-sAC signaling by acetazolamide, KH7 or H89 results in increased ROS production.

To identify which respiratory chain complex is the target of mito-sAC signaling, the author fueled the respiratory chain with complex-specific electron donors (glutamate/malate, succinate, and ascorbate/TMPD for complex I, II, IV respectively) and found that addition of KH7 and H89 invariably reduces respiratory chain activity, suggesting complex IV, cytochrome c oxidase (COX), is likely the target for PKA phosphorylation (Acin-Perez et al., 2011a). By PKA consensus site searching, Ser-85, located on the matrix side of the COX subunit IV-1 was identified as a possible PKA target in mito-sAC signaling. Protein mutagenesis at Ser-85 of COXIV-1 confirmed that Ser-85 is necessary for efficient oxidative phosphorylation. Furthermore, phosphorylation at Ser-85 of COXIV-1 relieves COX from allosteric inhibition by ATP, which further enhances respiratory chain activity.

Recently, the dynamic mitochondrial phosphoproteome has come to the center of metabolic regulation (Hopper et al., 2006; Pagliarini and Dixon, 2006; Grimsrud et al., 2012). The identification of a mito-sAC signaling pathway certainly provides a mechanism for phosphorylation of mitochondria proteins. Further research is needed to explore whether the mito-sAC pathway also regulates other metabolic processes in mitochondria.

### sAC is involved in glucose-stimulated insulin secretion (GSIS) of β-cells in the pancreas

Glucose homeostasis of mammals is largely regulated by the insulin secretion of β-cells and glucagon secretion of α-cells in pancreas. In the widely accepted model for coupling glucose sensing to insulin secretion by β-cells, increased plasma glucose levels (e.g., after meals) result in increased cytosolic glucose ([glucose]_c_), which leads to increased generation of ATP via glycolysis, Krebs cycle, and respiratory chain. The ATP derived from glucose metabolism then diffuses through the cytoplasm to the plasma membrane. Elevated local ATP/ADP ratio inhibits the ATP-sensitive complex of the sulfonylurea receptor (SUR) with its potassium channel (K_ATP_), which results in membrane depolarization and leads to opening of the voltage-dependent Ca^2+^ channel. The calcium influx thereby triggers the exocytosis of ready-docked insulin granules (Henquin, 2000). In addition to calcium influx, a concomitant rise in intracellular cAMP following glucose stimulation has been long noticed (Charles et al., 1973, 1975; Ammala et al., 1993; Dyachok et al., 2008); however, its role in GSIS was not completely understood. Ramos et al. demonstrated in the insulinoma cell line INS-1E that while the gut-derived incretin, glucagon-like peptide-1 (GLP-1), induced a rapid increase of intracellular cAMP via G protein-coupled receptor (GPCR) and tmAC, a direct glucose stimulus (16 mM) results in a delayed (detectable at least after 5 min) calcium-dependent rise of cAMP for which sAC is responsible (Ramos et al., 2008).

Elevation in [Glucose]_c_ likely activates sAC in a feed forward way. As ATP derived from increased [glucose]_c_ will diffuse away from mitochondria, the unique localization of sAC in mitochondria and cytosol lends itself ready access to its substrate. As demonstrated by Zippin et al. cellular sAC activity is dependent on intracellular ATP level (Zippin et al., 2013). Since activation of sAC facilitates glycolysis and enhances Krebs cycle-respiratory chain coupling, sAC might play an important role in GSIS in β-cells by making ATP generation more efficient. Consistently, in INS-1E cells, GSIS was inhibited by both KH7 and 2-hydroxyestradiol in a dose-dependent manner; knockdown with si-RNA against sAC resulted in ~50% decrease in protein expression and comparable reduction in insulin secretion (Zippin et al., 2013). However, the molecular mechanism of this sAC-dependent GSIS remains to be investigated. Interestingly, Zippin et al. also demonstrated that, though morphologically normal, isolated pancreatic islets from sAC C1KO mice had less insulin secretion compared with the wild-type mice when perifused with Krebs-Ringer bicarbonate buffer containing 30 mM glucose. Furthermore, when compared with the heterozygote mice (Sacy^tm1Lex/^Sacy^+^) in an peritoneal glucose tolerance test, the C1KO mice (Sacy^tm1Lex/^Sacy^tm1Lex^) showed an elevated maximal plasma glucose level, delayed glucose clearance, and reduced insulin release. This would suggest that the testicular isoforms of sAC (full-length and truncated), instead of the somatic isoform, is responsible for the regulation of GSIS.

Altogether, cAMP signaling by the HCO^−^_3_/CO_2_-sensing sAC constitutes an emergent regulatory mechanism in glucose metabolism and homeostasis. Importantly, the mito-sAC signaling accounts for the vital coupling between Krebs cycle and respiratory chain. However, it remains unclear how the glycolysis in cytosol is coupled to the Krebs cycle in the mitochondria. cAMP signaling is unlikely the missing link as it has been demonstrated recently that mitochondria inner membrane is impermeable to cytosolic cAMP (Acin-Perez et al., 2009; Lefkimmiatis et al., 2013). An alternative way to bridge the cytosol and mitochondrial matrix might be through Ca^2+^ signaling with sAC (Jaiswal and Conti, 2003; Litvin et al., 2003) together with a few Ca^2+^-sensitive dehydrogenase complexes (McCormack et al., 1990; McCormack and Denton, 1993) as potential downstream targets. This is supported by a recent study by Di Benedetto et al. demonstrating that mitochondrial Ca^2+^ uptake induces cAMP generation in the mitochondrial matrix and modulates ATP level (Di Benedetto et al., 2013).

## Other proposed functions of soluble adenylyl cyclase

### Regulation of CFTR by soluble adenylyl cyclase

It is well established that CFTR is activated by cAMP (Bridges, 2012). CFTR plays an important function in direct bicarbonate transport (by channeling HCO^−^_3_) and indirect bicarbonate transport (by stimulation of chloride/bicarbonate exchange); therefore the question can be raised whether cAMP derived from sAC plays a role in activation of CFTR. This would constitute a direct regulatory role of the bicarbonate sensor sAC in bicarbonate transport. Indirect evidence for this contention was provided by the observation of bicarbonate-induced phosphorylation of CFTR in corneal epithelial cells (Sun et al., 2003). Wang et al. (2005) observed a stimulation of CFTR-mediated whole cell currents by bicarbonate in human airway Calu-3 cells, which could be inhibited by 2-hydroxyestradiol, an inhibitor of sAC. In the same cell type Baudouin-Legros et al. (2008) observed a strong dependence of CFTR expression on the amount of bicarbonate in the culture medium. This dependence was associated with increased cAMP production as well as increased CREB phosphorylation. Both the CREB phosphorylation and the induction of CFTR by bicarbonate could be mitigated by 2-hydroxyestradiol, suggesting that sAC plays a crucial role in this regulation. Although further studies are clearly warranted these observations suggest that sAC may regulate CFTR function both at the transcriptional and at the posttranslational level.

### Apoptosis

There is a host of data in the literature demonstrating that changes in cellular cAMP levels influence apoptosis. The problem, however, is that much information is contradictory. Thus, cAMP has been reported to act both as a pro- and anti-apoptotic messenger. The insight has grown that conflicting results may in part be related to the compartmentation of cAMP signaling. In addition, similar interventions have been found to cause very different effects in different cell lines. Recently, the distinction between cAMP generated by transmembrane AC's vs. cAMP generated by sAC has provided some new information that may help to explain these controversies.

The group of Ladilov (Kumar et al., 2009) has studied the role of sAC in apoptosis in endothelial cells and found that sAC translocates to mitochondria under ischemic acidosis. They further showed that sAC inhibition with KH7 or sAC knockdown abolished the activation of the mitochondrial pathway of apoptosis. Analysis of mitochondrial co-localization of Bcl-2 family proteins demonstrated sAC- and protein kinase A-dependent translocation of Bax to mitochondria. Taken together, these results suggest the important role of sAC in activation of the mitochondria-dependent pathway of apoptosis in endothelial cells. However, at the same time these authors observed an inhibition of apoptosis by forskolin treatment. Hence, cAMP coming from transmembrane adenylyl cyclases may have an opposite effect to cAMP generated by sAC. In a more recent publication (Appukuttan et al., 2012) the same group recently showed similar results for ischemia-induced apoptosis in cardiomyocytes. In this report they additionally demonstrated that inhibition of sAC with KH7 prevents phosphorylation of Bax at Thr167 as well as mitochondrial Bax translocation, suggesting that sAC triggers this process. How mitochondrial sAC would become activated during an apoptotic trigger is unknown. It is interesting to note that the group of Ladilov (Kumar et al., 2011) reported recently that ischemic apoptosis in endothelial cells is enhanced by inhibition of the sodium bicarbonate transporter SLC4A7, which they partially localized to mitochondria. Hence, dysregulation of intramitochondrial bicarbonate concentration may play a regulatory role in the apoptotic trigger.

At present, it is far from clear whether it is mitochondrial sAC that is a general key step in the trigger for mitochondrial apoptosis. The Ladilov group (Appukuttan et al., 2013), very recently, studied oxysterol-induced apoptosis in smooth muscle cells. In these studies, it was confirmed that apoptosis can be inhibited by KH7, as is the case for endothelial cells and cardiomyocytes. However, upon specific targeting of sAC to mitochondria or cytosol, the authors observed a stronger apoptotic trigger with cytosolic sAC than with mitochondrial sAC, suggesting that the signal must come from the cytosol.

### Barrier function

In many studies it has been reported that cAMP generated by transmembrane ACs strengthens the barrier through activation of plasma membrane targets, such as the actin binding protein, filamin. This cAMP pool is confined to its subplasma membrane compartment by diffusion barricades, such as PDE activity, which hydrolyzes cAMP before it can escape to the cytosolic compartment.

A few studies have shown that when cAMP gains access to or is generated within the cytosolic compartment it has an opposite signaling effect, namely barrier disruption. Sayner et al. (2006) addressed this apparent discrepancy by targeting an adenylyl cyclase either to the plasma membrane or to the cytosol of pulmonary endothelial cells. Their primary goal was to explain the fact that cAMP from tmAC's leads to strengthening of the endothelial barrier, whereas pathogenic bacteria insert soluble adenylyl cyclases (such as ExoY from *P. aeruginosa*) into eukaryotic cells generating an increase in cytosolic cAMP that disrupts the endothelial barrier. Transduction of cells with an artificial AC construct that gives rise to adenylyl cyclase activity in the cytosol and subsequent stimulation of this AC by forskolin led to a decreased barrier function through the formation of gaps. Gap formation was not observed when the same construct was targeted to the plasma membrane. In a recent study Obiako et al. (2013) they showed that pulmonary microvascular endothelial cells (PMVECs) express much higher levels of soluble adenylyl cyclase than pulmonary artery endothelial cells (PAECs). They also showed that incubation with bicarbonate increased cAMP in PMVECs but not in PAECs and that under these conditions PMVECs had lower barrier function than PAECs. This might be interpreted as indirect evidence for a disruptive effect of cAMP generated by sAC in the cytosol as opposed to the strengthening effect of subplasma membrane cAMP.

## Concluding remark

The bicarbonate-responsive soluble adenylyl cyclase is an emergent sensor and regulator in pH homeostasis and a coupler for various metabolic processes. In concert with CA, both pH and CO_2_ are translated to bicarbonate as input signal. Ca^2+^ serves as a fine-tuner for soluble adenylyl cyclase and provide a window for crosstalk. The output, cAMP, is the most versatile second messenger with several downstream effectors capable of short-term and long-term regulation. Further investigation will surely gain us more insight into how this evolutionarily conserved enzyme takes part in homeostasis of pH and cellular metabolism.

### Conflict of interest statement

The authors declare that the research was conducted in the absence of any commercial or financial relationships that could be construed as a potential conflict of interest.

## Appendix

**Table:**

**Organ**	**Cell type/cell line/organelle**	**Function of sAC**
Testis	Spermatocytes	Spermatogenesis, maturation of sperms in epididymis, capacitation, fertilization^*^
Epididymis	Clear cells	Maintain acidic luminal pH via apical insertion and endocytosis of V-ATPase^*^^†^
Kidney	Alpha-intercalated cells in collecting ducts	Regulate apical proton secretion in response to luminal bicarbonate via apical insertion and endocytosis of V-ATPase^*^^†^
Brain	Astrocytes in hippocampus	Glycogenolysis, glycolysis, lactate production
Brain and liver	Mitochondria	Couple Krebs cycle to respiratory chain for efficient utilization of reducing equivalents and oxidative phosphorylation^*^
Endocrine pancreas	β-cells	Glucose-stimulated insulin secretion
N/A	Calu-3 human lung adenocarcinoma cell line	CFTR activity and expression
Heart	Coronary endothelial cells Cardiomyocytes	Pro-apoptotic. Inhibition of sAC reduces Bax Thr167 phosphorylation, mitochondrial Bax translocation and mitochondrial-dependent apoptosis^*^
Lung	Pulmonary microvascular endothelial cells	Regulation of barrier function. Exposure to high bicarbonate is associated with decreased resistance across the monolayer

* *Mediated by protein kinase A*.

† *Counteracted by AMP-activated protein kinase*.

*N/A, not applicable*.
